# Design and Analysis of a Bézier Curve-Based Variable Cross-Section Magnetoelectric Antenna

**DOI:** 10.3390/ma19153335

**Published:** 2026-08-05

**Authors:** Gang Li, Naijun Zhao, Jiangang Li, Xin Ma, Shipeng Liu, Guoxuan Zhang, Shiren La, Yang Shi, Qiyuan Jiao

**Affiliations:** 1 Qinghai Third Road and Bridge Construction Co., Ltd., Xining 810001, China; 2 School of Mechano-Electronic Engineering, Xidian University, Xi’an 710071, Chinashiyang@xidian.edu.cn (Y.S.);; 3 School of Civil and Transportation Engineering, Qinghai Minzu University, Xining 810007, China

**Keywords:** ME antenna, multi-physics coupling, variable-cross-section structure, shape control factor

## Abstract

Conventional low-frequency antennas face a trade-off between miniaturization and radiation efficiency due to wavelength limitations. Although magnetoelectric (ME) antennas can overcome the electrical size constraint, existing designs lack structural tunability and performance enhancement. This paper proposes a Bézier curve-based (BCB) ME antenna that features a variable cross-section, introducing a shape tuning factor for precise geometric configuration. Using the lumped-mass method, the functional relationship between resonant frequency and the shape tuning factor is derived, establishing the theoretical basis for frequency tuning. A nonlinear multi-field coupled numerical simulation model is established for performance prediction. The BCB structure modifies internal stress distribution, enabling spatial reconstruction of magnetization modulation. The proposed design is validated by comparing the analytical model with our simulation results and literature-reported experimental data. Results show that the BCB design reduces resonant frequency and enhances converse ME (CME) coupling and far-field radiation without increasing material volume. Under clamped and free boundary conditions, the minimum resonant frequencies reach 7.2 kHz and 11.1 kHz, respectively, with CME coupling improved by 124% and 140%. When the shape tuning factor proposed in this work is set to 1/2, the proposed design degenerates into a traditional antenna with uniform cross-sections, which verifies the consistency of the established model.

## 1. Introduction

An antenna is a core device that converts signal electrical energy into spatial electromagnetic (EM) waves [1]. They are now widely used in smartphones, wearable devices, and various portable terminals [2,3]. Conventional antennas rely on conductive currents for radiation, and their dimensions must reach at least one-tenth of the wavelength to ensure efficiency [4,5]. This physical constraint forces low-frequency and very-low-frequency (3 kHz–30 kHz) antennas to be kilometer-scale, making them impossible to integrate into miniature devices or apply in extreme environments. To fundamentally decouple the antenna size from the operating wavelength, magnetoelectric (ME) antennas have emerged. They exploit the multi-field coupling of magnetostrictive and piezoelectric materials to generate an equivalent magnetic current through mechanical vibration, rather than relying on conductive current radiation [6]. This new mechanism reduces antenna dimensions to as small as one ten-thousandth of the wavelength or even less, while being compatible with micro-nanofabrication processes, promising a shift from device-level to chip-scale integration.

With the conceptual proposal [7,8] and experimental realization of ME antennas, research focus has gradually shifted to performance analysis and structural optimization, as well as the exploration of engineering applications such as energy harvesting [9,10,11], magnetic sensing [12,13,14], and low-frequency communication [15,16,17]. In numerical analysis, the modeling evolution has progressed from global equivalent models to structure-level coupled analysis, and further to nonlinear multi-field refined modeling. Early efforts aimed at a unified description of acoustic wave propagation and EM response. Yao et al. [7] introduced a one-dimensional multi-scale finite-difference time-domain method to jointly predict acoustic resonant modes and radiation behavior, establishing a preliminary propagation model; subsequently, they proposed a set of governing equations coupling EM waves, magnetic spin, and acoustics [8]. However, these models are based on one-dimensional or uniform-field assumptions and thus cannot accurately describe complex geometric boundaries, local stresses, or internal field reconstruction. To more precisely characterize radiation characteristics, researchers have developed structure-level coupled analysis models. For example, Truong et al. [18] provided an analytical solution for wireless power transfer systems, which began to handle realistic structures and boundaries, but mainly focused on static ME coupling.

To improve the accuracy of magnetization dynamics analysis, Xu et al. [19] introduced the Landau–Lifshitz–Gilbert equation to study magnetoelastic coupling; Bickford et al. [20] analyzed the miniaturization bottleneck of conventional electrically small antennas from a magnetic dipole perspective; Du et al. [21] experimentally extracted the effective magnetic dipole moment; and Domann et al. [22] established a unified multi-physics framework and conducted micromagnetic simulations. Subsequently, Xie et al. [23] derived near-field coupling equations, and Xu et al. [24] solved the near-field radiation performance, gradually completing the theoretical chain from stress/strain to magnetostriction-induced radiation. However, the understanding of non-uniform stress fields and nonlinear magnetoelastic coupling remains insufficient. Studies have shown that magnetostrictive materials exhibit strongly nonlinear responses under bias magnetic fields and pre-stresses [25,26]. Du et al. [27] further proposed a two-dimensional multi-field coupling model that accounts for material nonlinearity and arbitrary excitation directions. To enhance the realism and predictability of models, multi-field coupling analysis methods have gradually become core tools [28], compensating for the inability of analytical models to reveal local field reconstruction. Rostami et al. [29] adopted a nonlinear isotropic model in COMSOL Multiphysics but linearized it in radio-frequency simulations. Luong et al. [30] incorporated a nonlinear constitutive law into a finite element model, moving modeling toward realistic nonlinear descriptions. Schneider et al. [31] found that magnetic antennas exhibit better propagation characteristics in lossy media. Chu et al. [32] studied the nonlinear behavior of ME heterostructures using the Stoner–Wohlfarth model and the Duffing equation. Lei et al. [33] developed a multi-field coupling radiation model based on a nonlinear magnetization constitutive law, revealing the strain/stress-mediated radiation enhancement mechanism. Subsequent studies confirmed that incorporating a nonlinear magnetostrictive constitutive law significantly improves prediction accuracy [34,35,36]. Overall, existing models have continuously improved in accuracy and completeness, but the methodological system remains dominated by two major approaches: FDTD and finite element simulations. How to balance computational efficiency, nonlinear fidelity, and adaptability to complex structures remains a critical challenge in the current modeling of ME antennas.

Building on numerical simulations, structural optimization of ME antennas has become an important means to enhance radiation performance and broaden bandwidth. In terms of geometric design, to lower the resonance frequency, Ma et al. [37] investigated a dumbbell-shaped ME composite structure and analyzed the influence of end-edge width on charge response. However, because acoustic resonance is confined near the natural frequency [38,39,40], this structure is limited in broadband applications. Min et al. [41] designed a fishtail-shaped ME resonator and verified its effectiveness in reducing resonance frequency and improving the ME coupling coefficient using finite element simulations and the lumped-mass method. Zaeimbashi et al. [42] proposed an ultra-miniaturized wireless implantable device capable of harvesting EM energy for power supply and detecting quasi-static neural magnetic fields as low as 200 fT with low tissue loss. Cheng et al. [43] developed a bio-inspired flapping-wing magnetic dipole resonator for cross-medium communication, significantly enhancing magnetic field emission capability; subsequently, they reported a centimeter-scale wearable ultrasound-driven magnetic dipole rotating resonator [44] to mitigate energy attenuation in biological media. Since then, research on implantable ME antennas has gradually increased [45,46,47], but most studies remain at the stage of ex vivo tissue-substitute experiments. Further investigations are still needed in terms of standard frequency adaptation, deep implantation, miniaturization, and detuning resilience. For material and interface optimization, Chen et al. [48] suppressed eddy current losses by inserting an Al_2_O_3_ thin film into FeGaB. Shi et al. [49] designed an embedded antenna for material and interface optimization, which improved near-field radiation performance by enhancing interfacial strain transfer. The structural design of ME antennas is continuously evolving toward greater diversity and refinement. Zhang et al. [50] designed a curved cantilever–beam mode ME antenna featuring high radiation intensity and a size reduction of over 95%, capable of operating at an ultra-low frequency of 193 Hz. Dong et al. [51] designed a strain-mediated, high-Q Metglas/Quartz very-low-frequency ME resonator that successfully received signals from the very-low-frequency transmitter at a distance of approximately 400 km with a signal-to-noise ratio of 55 dB. Deng et al. [52] exploited the self-bias field induced by hysteresis effects and achieved strong radiation output without an external magnetic field through material modification, greatly broadening engineering application scenarios. These innovative designs have laid an important foundation for the application of ME antennas in areas such as high-sensitivity detection, very-low-frequency transmission, and wireless implantable devices.

According to existing research analysis, ME antennas have achieved significant progress in size miniaturization and multi-frequency design, offering a new technical pathway to overcome the size–efficiency trade-off of conventional electrically small antennas. However, from an engineering application perspective, there remains room for optimizing the magnetization modulation efficiency of current ME antennas. At present, most ME antennas adopt uniform-cross-section (UCS) bulk structures, and their theoretical analyses and numerical models generally rely on the assumptions of approximately uniform stress distribution and uniform magnetization modulation. In an ME structure, the mechanical strain generated by the piezoelectric phase is transferred to the magnetostrictive layer through interface coupling, inducing time-varying oscillations of magnetization and forming an equivalent magnetic dipole moment. From the perspective of radiation, the far-field intensity depends on the spatial integral effect of magnetization, i.e., the time-varying amplitude of the magnetic dipole moment. However, uniform structures lack the ability to actively manipulate field distributions at the geometric level; the stress and magnetic field distributions are primarily determined by the intrinsic material parameters and remain relatively smooth. Consequently, the amplitude of magnetization modulation is averaged over the volume, limiting the peak response of the magnetic dipole moment. To address this issue, this paper introduces a Bézier curve-based (BCB) ME structure with variable cross-sections (VCS). By exploiting the stress concentration effect induced by geometric gradients, the spatial distribution of magnetization modulation within the magnetostrictive layer is reconfigured, thereby enhancing the effective oscillation amplitude of the equivalent magnetic dipole moment. The remainder of this paper is organized as follows: Section 2 describes the design of the BCB ME antenna and introduces a shape control factor for precise geometric adjustment. Section 3 develops a lumped-element model and a multi-field coupling analysis model to determine the tunable range of the resonance frequency and performs systematic simulation analyses. Section 4 validates the reliability of the computational model and demonstrates the performance enhancement achieved by the optimized antenna. Section 5 concludes the paper.

## 2. Design of the BCB ME Antenna

In this section, a BCB ME antenna featuring a variable cross-section is proposed. By adjusting the antenna geometry, the BCB structure can exploit the stress concentration effect to enhance the converse ME (CME) effect, thereby lowering the resonant frequency while improving radiation performance. Specifically, the BCB design, through geometric optimization, is expected to reduce the resonance frequency of the longitudinal vibration mode of the ME resonator and simultaneously improve its coupling performance. In this study, a nonlinear multi-physics coupling model is employed, combined with finite element simulations and the lumped-mass method, to investigate how the antenna structural modification reduces the resonant frequency and to analyze the underlying mechanisms responsible for the enhanced coupling performance.

### 2.1. BCB ME Antenna Structure

**Figure 1:**
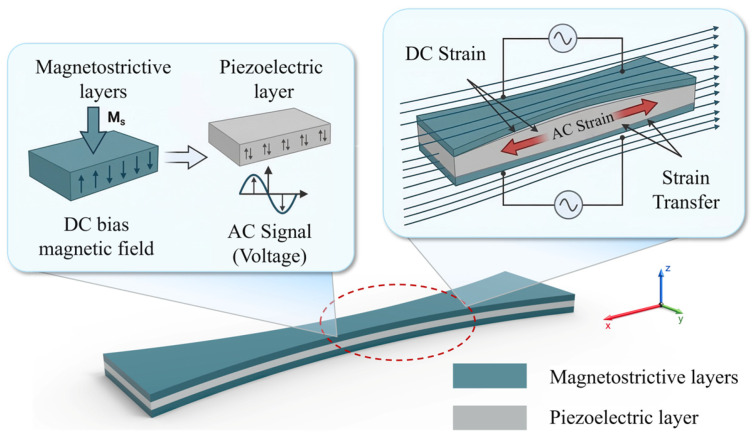
Configuration and working principle of the BCB ME antenna.

Figure 1 shows the structural schematic of the BCB ME antenna. This layered composite structure consists primarily of a magnetostrictive layer, a piezoelectric layer, and electrode layers, which are bonded together using epoxy resin to form interfacial coupling (the electrode layers are attached to the upper and lower sides of the piezoelectric layer). The ME antenna exploits the coupling effect between the magnetization of the magnetostrictive material and the polarization of the piezoelectric material to achieve EM radiation. During transmission, an alternating current (AC) voltage is applied to the electrodes on both sides of the piezoelectric layer, inducing forced vibration through the piezoelectric effect. This vibration is transmitted through the bonding layer to the magnetostrictive layer, where it induces magnetization oscillation within the magnetostrictive material under a bias magnetic field. As a result of the magnetization oscillation, a dynamic magnetic flux is generated inside the magnetostrictive layer. This dynamic magnetic flux, in turn, induces a dynamic electric field at the surface of the magnetostrictive layer, producing an aperture field that radiates EM waves. The proposed BCB ME antenna operates in the L-T mode (the piezoelectric layer is polarized along the z-axis, and the magnetic layer is magnetized along the x-axis). Terfenol-D, PZT-5H, and Al are selected as the materials for the magnetostrictive layer, piezoelectric layer, and electrode layers, respectively. The main parameters of these materials are listed in the Supplementary Materials. In the designed ME antenna, the width at both ends remains constant, while the width gradually decreases from the ends toward the middle along the length direction, forming a smooth curved cross-sectional profile. The antenna has an overall length l, thickness h, and a fixed width w at both ends. A Cartesian coordinate system is established with its origin at the midpoint of the central layer (in the thickness direction) of the antenna structure, where the x-axis, y-axis, and z-axis are aligned with the length, width, and thickness directions, respectively. To systematically characterize the variation of the cross-sectional geometry, a shape control factor is introduced to parametrically represent and adjust the VCS profile of the antenna.

### 2.2. Cross-Sectional Shape Factor

In this section, Bézier curves are used to control the gradient variation of the cross-section of the ME antenna. The main reasons for choosing this type of curve are twofold: (1) Bézier curves can generate continuous, smooth, and adjustable profiles and are widely used in structural shape design; (2) mainstream simulation software such as COMSOL Multiphysics includes a built-in three-point quadratic Bézier curve module, which facilitates direct implementation and modeling. In general, a Bézier curve can be described by the following equation:

(1) $$B(\tau )={\left(1-\tau \right)}^{n}P_{0}+n\tau {\left(1-\tau \right)}^{n-1}P_{1}+\cdots +\tau^{n}P_{n}\;(0\leq \tau \leq 1)$$

where B(τ) is the point on the Bézier curve at parameter τ, $P_{0-n}$ describes the number of control points of the Bézier curve and determines the shape of the curve, and n is the degree of the Bézier curve. As the degree increases, the expressive power and complexity of the curve also increase, enabling more flexible and precise shape control. In this study, a three-point quadratic Bézier curve is adopted, described by the following:

(2) $$B(\tau )={\left(1-\tau \right)}^{2}P_{0}+2(1-\tau )\tau P_{1}+\tau^{2}P_{2}\;(0\leq \tau \leq 1)$$

**Figure 2:**
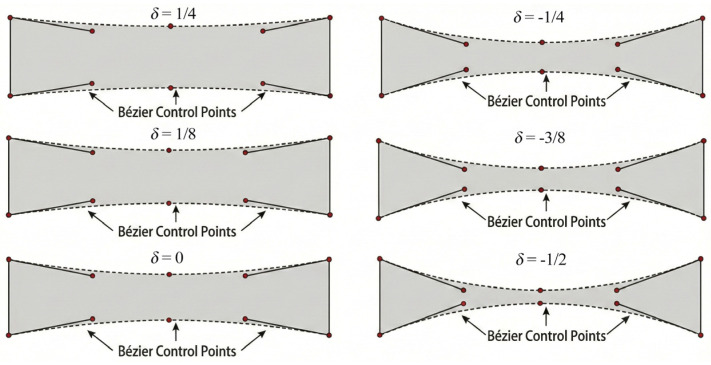
Design mechanism of the BCB ME antenna: top views of the structure for different δ values.

As τ varies from 0 to 1, the curve smoothly transitions from the start point $P_{0}\;$ to the end point $P_{2}\;$ and is influenced by the control point $P_{1}\;$, which determines the degree of curvature. By adjusting the Bézier curve in Equation (2), the BCB antenna can be designed. Specifically, the two endpoints of the curve (i.e., the two ends of the antenna) are set as $P_{0}=\;(-l/2,\;w/2)$ $P_{2}=\;(l/2,\;w/2)$, ensuring that both endpoints lie at the same horizontal level. The intermediate control point is $P_{1}\;=\;(0,\;w_{c})$, where $w_{c}$ is an adjustable dimensional parameter that controls the curvature of the curve. By varying the value of $w_{c}$, the overall profile of the antenna cross-section can be flexibly adjusted while keeping the total length and total width unchanged, thereby optimizing its EM radiation performance. Substituting the fixed coordinates of $P_{0}$, $P_{1}$, and $P_{2}$ into the three-point quadratic Bézier curve expression and simplifying, the vertical coordinate *y* of the curve can be expressed as an explicit function of the horizontal coordinate *x*:

(3) $$y(x)=\frac{w}{2}+\frac{w}{2}\left(\delta -\frac{1}{2}\right)\left[1-{\left(\frac{2x}{l}\right)}^{2}\right]\;\;-\frac{l}{2}\leq x\leq \frac{l}{2}$$

where $\delta =w_{c}/w$ is a dimensionless parameter with a theoretical range of [−1/2, 1/2] in this study, and δ is defined as the shape control factor. When δ=1/2, the VCS antenna degenerates into a conventional UCS structure with a constant width w; when δ=−1/2, the width at the center of the antenna theoretically approaches zero. Figure 2 shows the top views of the BCB ME antenna corresponding to different values of the shape control factor δ. The results indicate that as δ decreases, the overall volume of the antenna tends to gradually decrease.

## 3. Numerical Analysis of the BCB ME Antenna

COMSOL Multiphysics 6.0 is used to perform numerical simulations of the BCB ME antenna in order to investigate its operating mechanism and radiation characteristics under multi-field coupling. First, the geometric model is established. Since each thin-film layer possesses a planar plate shape, the modeling is mainly realized via sketching and extrusion operations. For variable cross-section designs, the final configuration is governed by Bézier curves (Equation (3)) with different shape factors. Moreover, a spherical volume with a specific thickness is constructed to serve as the infinite element domain for simulating an unbounded region. Subsequently, physical field conditions are configured. The simulation primarily involves three physical modules, “Electrostatics (es)”, “Magnetic Fields (mf)”, and “Solid Mechanics”, with the piezoelectric and magnetostrictive effects being co-simulated through multi-physics coupling. Specifically, the piezoelectric material realizes the piezoelectric effect via the coupling of the electrostatic field and the solid mechanics field, while the magnetostrictive material describes the magnetostrictive magnetization behavior through the coupling of the magnetic field and the solid mechanics field.

In previous finite element simulation analyses of ME composite structures, separate static and dynamic loading modes were commonly used to simulate the ME effect under DC and AC electric fields [53,54]. However, an ME antenna in actual operation requires the simultaneous application of a DC bias magnetic field and an AC excitation: the former is used to fully magnetize the magnetostrictive layer, ensuring a significant magnetostrictive effect, while the latter excites the dynamic response of the resonator and drives the ME coupling process. To describe this process, the “Small Signal Analysis, Frequency Domain” study type is selected to investigate small-amplitude oscillations about a bias solution. The specific simulation procedure is as follows [55]:

(1) DC simulation is performed using a stationary solver, involving the Magnetic Fields and Solid Mechanics modules. Under the applied DC bias magnetic field, the magnetostrictive layer exhibits the Villari effect. The effective magnetic field in the material is computed using the nonlinear constitutive equation of the magnetostrictive material [34]. At this stage, the magnetostrictive layer is in a magnetized state. Given that the built-in magnetostrictive model in finite element software cannot fully describe the nonlinear force-magnetic coupling behavior, a numerical iterative method is required to solve the nonlinear magnetization equation, which is then imported into the finite element model as a material property parameter. Subsequently, by coupling the magnetic field and solid mechanics equations, the magnetization distribution in the magnetostrictive layer and the corresponding magnetostrictive strain under the combined action of the DC bias magnetic field and pre-stress are calculated. This result provides a stable initial operating point for the subsequent AC simulation.
(2) AC simulation is carried out using a frequency-domain perturbation solver, coupling the Electrostatics, Magnetic Fields, and Solid Mechanics modules. In the small-signal frequency-domain perturbation analysis, the frequency-domain perturbation range is set to 0–11 kHz based on the results of the lumped mass method. During the perturbation, an AC voltage of 1 mV is applied to the upper and lower electrodes of the piezoelectric layer. The piezoelectric layer generates dynamic strain through the piezoelectric effect, which is realized via the piezoelectric material constitutive equations, electrostatic equations, and dynamic governing equations. This dynamic strain is then transmitted through the interface coupling to the already magnetized magnetostrictive layer, inducing dynamic strain in the magnetostrictive layer via the inverse magnetostrictive effect, described by the nonlinear magnetostrictive constitutive equations and dynamic governing equations. Through this process, the Maxwell equations in the magnetic physics interface are solved to obtain the dynamic magnetization generated in the magnetostrictive layer.
(3) The dynamic magnetic flux obtained in the previous step serves as a point radiation source. By integrating the magnetic flux density over the magnetostrictive layer, the magnetic dipole moment inside the radiating layer is calculated. Subsequently, the far-field radiation of the antenna is determined using the radiation field theoretical model.

**Figure 3:**
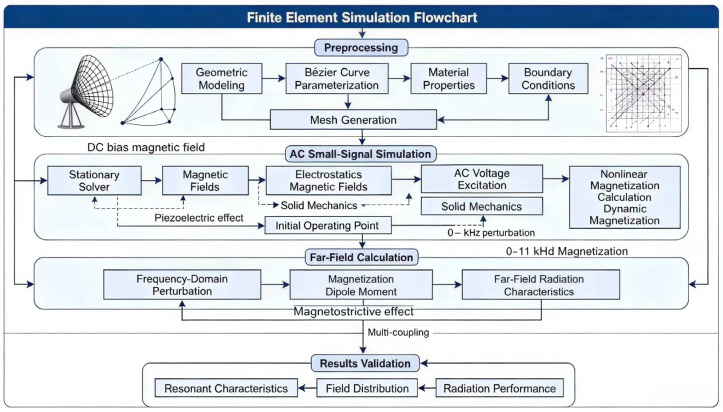
Multi-field coupling simulation process.

In the finite element simulation, an air domain with a radius of 630 mm is defined around the ME structure to simulate a realistic external environment and to analyze EM radiation. Regarding mesh generation, free tetrahedral meshes are adopted for the functional layers of the model, while swept meshing is implemented for the infinite element domain to reduce computational cost and enhance simulation efficiency. The element size is set to Normal, with a maximum element size of 126 mm, a minimum element size of 22.7 mm, a maximum element growth rate of 1.5, a curvature factor of 0.6, and a narrow region resolution of 0.5. Taking the resonant frequency as the objective, a mesh independence study is carried out, and the final mesh number is determined to be approximately 15,979. In terms of boundary settings, within the Electrostatics module, terminal and ground boundary conditions are assigned to the upper and lower surfaces of the piezoelectric layer, respectively. Both cantilever and free-standing structures are investigated (see Supplementary Materials for details). The multi-field coupling simulation process of the BCB ME antenna is shown in Figure 3.

**Figure 4:**
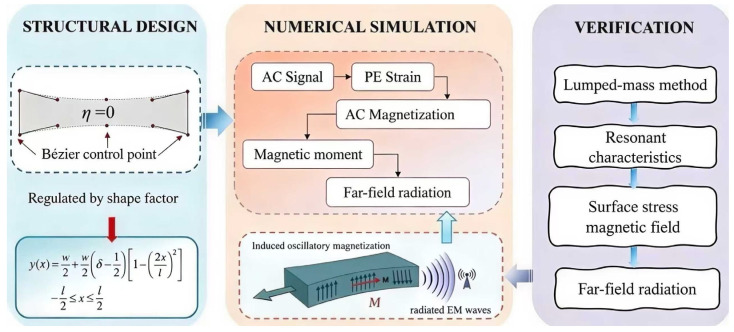
Flowchart of the design, simulation, and verification process for the BCB ME antenna.

During signal transmission, the ME antenna relies on the CME effect to convert electrical signals into EM radiation. To quantitatively describe this electro-magnetic coupling capability, the CME coefficient $\alpha_{CME}=\left|\delta H/\delta E\right|$ is introduced as a key characterization parameter, where δH is the average AC magnetic field induced in the radiating layer and δE is the average AC electric field induced in the piezoelectric layer. The CME coefficient reflects the ability to generate a magnetic response per unit electrical excitation; a larger value indicates a stronger modulation capability of the electric field on magnetization and a higher conversion efficiency from electrical energy to EM radiation energy. After constructing the finite element model, the proposed design is validated in multiple dimensions by calculating resonant characteristics, internal field distributions, and far-field radiation. The design, simulation, and verification process for the VCS ME antenna is illustrated in Figure 4.

## 4. Results and Discussion

### 4.1. Validation of the Theoretical Model

To verify the generality and predictive capability of the proposed analytical model for the BCB ME antenna, we applied it to predict the performance of two structures reported in ref. [41]: a conventional rectangular resonator and an F-L type fishtail resonator. All experiments were conducted under a uniform bias magnetic field of 10 mOe without pre-stress. To assess the reliability of our model, all simulation parameters were set to strictly replicate the experimental conditions, and the impedance characteristics of both structures were simulated.

**Figure 5:**
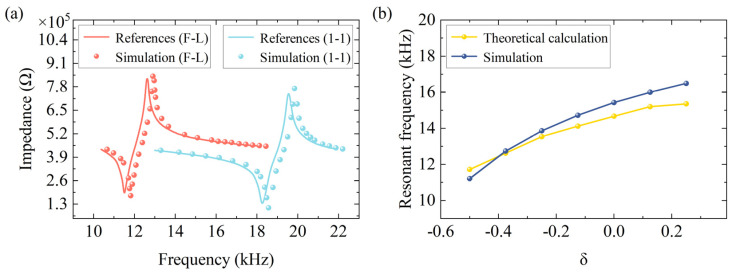
Performance comparison of the VCS ME antenna: (**a**) Impedance characteristics and (**b**) resonance frequency.

Figure 5a compares finite element simulations and the published experimental data. Overall, the impedance characteristics of both the conventional rectangular and F-L type fishtail resonators show reasonable consistency between simulation and experiment, which verifies that the established model is capable of characterizing the vibration behavior of the proposed antenna. Nevertheless, slight discrepancies between numerical simulations and experimental results can be noticed in terms of resonant frequency and peak impedance. Such deviations mainly arise from the omission of the epoxy bonding layer in the simulation setup. Serving as the adhesive between ME composite layers, the epoxy layer introduces dielectric loading effects and interfacial mechanical losses, leading to resonant frequency shifts and degraded ME coupling efficiency. To further validate the model’s effectiveness, we compared the resonance frequencies of the system calculated using the lumped-mass method [56,57] with those obtained from finite element simulations, as shown in Figure 5b. The resonant frequency determines the effective operating frequency band and judges whether the structural dimensions match the target operating frequency. The lumped-mass method, which is used to preliminarily evaluate the resonant frequency of the BCB ME antenna, is detailed in the Supplementary Materials. The results demonstrate that, under different tuning factors, the theoretical calculations and simulation results are in excellent agreement, which further corroborates the reliability of the proposed model.

In the antenna configuration of this work, the epoxy bonding layer matches the planar area of the piezoelectric and magnetostrictive functional layers, with a constant thickness far smaller than that of the functional layers. Under such structural characteristics, adjusting the antenna’s structural parameters changes the volume fraction of epoxy within the overall composite, which further governs the variation trend of modelling error: enlarging structural parameters reduces the volume fraction of epoxy and gradually lowers the modelling error, while reducing structural parameters increases epoxy volume fraction and leads to larger modelling deviations. Accordingly, when the volume fraction of epoxy in the composite structure becomes sufficiently high such that its mechanical and dielectric influences cannot be ignored, an elastic interlayer module corresponding to the epoxy adhesive should be incorporated into the theoretical model to correct this error source and further improve computational accuracy.

### 4.2. Resonant Characteristics and ME Effect

During the interfacial coupling between the magnetostrictive layer and the piezoelectric layer, the stress and strain distributions within the material directly influence the energy conversion efficiency and the overall radiation performance. When the material is subjected to external forces, the local stress field not only modulates the polarization state of the piezoelectric layer but also alters the magnetization direction of the magnetostrictive layer. This stress-driven ME effect enhances the ME coupling and thereby improves antenna performance. Therefore, investigating the stress distribution within the ME composite material is essential for a deeper understanding of its vibration characteristics, resonant frequency, and ME coupling behavior. In the subsequent study, the total length is set to *l* = 70 mm, the width to *w* = 16 mm, the thicknesses of both the magnetic and piezoelectric layers to 1 mm, and the thickness of the electrode layer to 0.1 mm.

**Figure 6:**
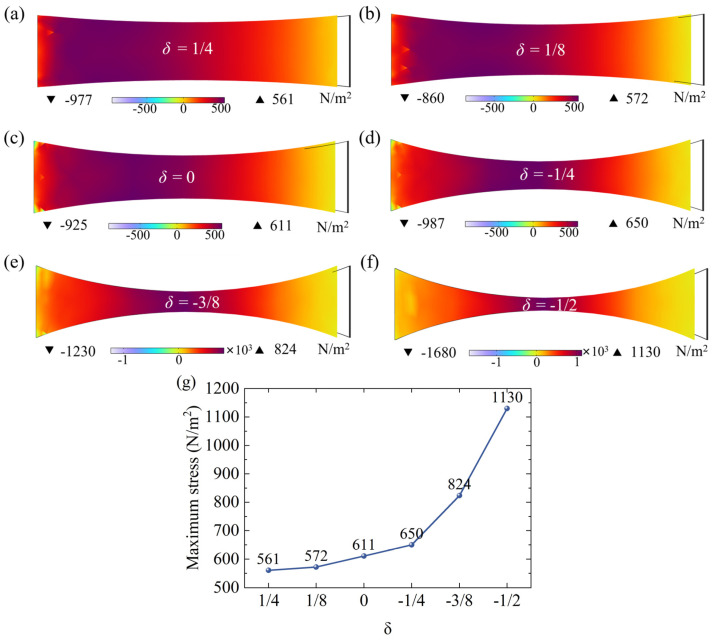
Stress distribution contours on the upper surface of the cantilever structure under different control factors: (**a**) δ = 1/4; (**b**) δ = 1/8; (**c**) δ = 0; (**d**) δ = −1/4; (**e**) δ = −3/8; (**f**) δ = −1/2; (**g**) Maximum stress vs. δ.

**Figure 7:**
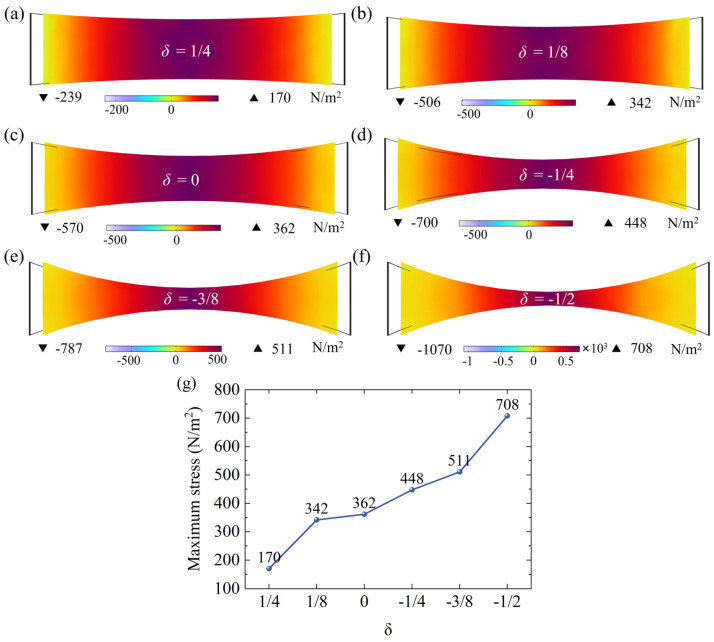
Stress distribution contours on the upper surface of the free structure under different control factors: (**a**) δ = 1/4; (**b**) δ = 1/8; (**c**) δ = 0; (**d**) δ = −1/4; (**e**) δ = −3/8; (**f**) δ = −1/2; (**g**) Maximum stress vs. δ.

Figure 6 shows the stress distribution contours of the cantilever structure under resonant modes. In the contours, negative values indicate compressive stress, while positive values indicate tensile stress. For example, when the control factor δ = 1/4, the contour shows a stress range from −977 to 561 ${N/m}^{2}$. The free end of the antenna exhibits tensile stress, indicating that this region bears a relatively high external load. As δ varies, the volume stress of the BCB ME antenna remains generally symmetric about the XOY plane. However, due to the fixed end of the antenna, the stress distribution along the x-axis exhibits obvious inhomogeneity. Further analysis reveals that as δ gradually decreases, the equivalent area participating in resonance also decreases, leading to a gradual increase in the maximum stress of the system. This trend has also been verified through simulations of the free structure. Unlike the cantilever structure, the free structure has no fixed constraints, and its stress distribution is approximately symmetric from left to right, reaching a maximum value at the center of the structure (see Figure 7). Based on the above analysis, the stress concentration phenomenon observed in the BCB antenna may potentially improve the ME conversion efficiency, a feature that is difficult to achieve with UCS antennas.

**Figure 8:**
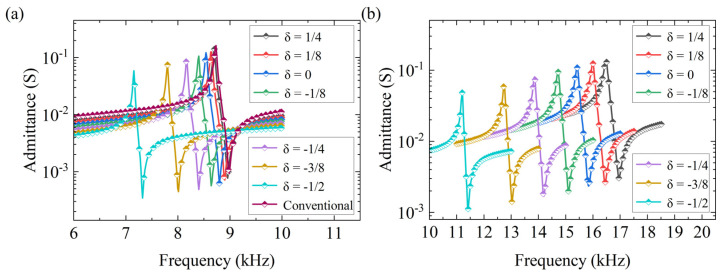
Resonant characteristics of the ME antenna as a function of different control factors: (**a**) Cantilever structure and (**b**) free structure.

**Figure 9:**
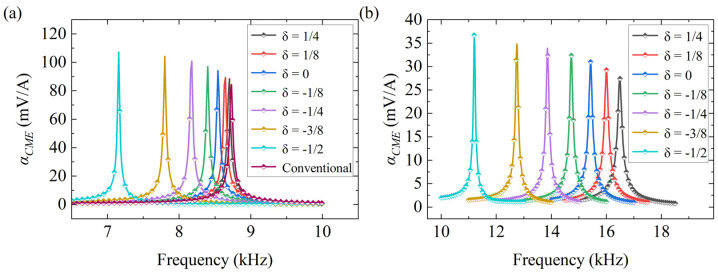
CME coefficient of the ME antenna under different control factors: (**a**) Cantilever structure and (**b**) free structure.

Figure 8 and Figure 9 illustrate the resonant characteristics and ME coupling performance of the BCB ME antenna under different control factors. It can be observed that as the control factor decreases, the resonant frequency of the system under both modes significantly decreases, while the ME coupling performance continuously improves. This indicates that adjusting the control factor can effectively alter the vibration behavior and energy coupling process of the antenna, demonstrating the significant regulatory effect of the BCB structural parameters on device performance.

Compared with conventional ME composite thin films, the resonant frequency of the cantilever structure at a scale of 70 mm decreases from 9 kHz to 7.2 kHz, while that of the free structure decreases from 18.1 kHz to 11.1 kHz. In the case study of this section, the lowest anti-resonance frequency of the ME resonator with a length of 120 mm reported in ref. [41] is 12.2 kHz. The BCB antenna achieves a lower resonant frequency at a smaller scale, highlighting its distinct advantage in low-frequency miniaturization design. Furthermore, at lower resonant frequencies, the cantilever structure and the free structure generate strong CME coupling coefficients of 109 mV/A and 33 mV/A, respectively. It is worth noting that under the same control factor, the resonant frequency of the cantilever structure is significantly lower than that of the free structure. For example, when the control factor δ = −1/8, the resonant frequency of the cantilever structure is 7.5 kHz, whereas that of the free structure is 14.5 kHz. This indicates that the fixed-end constraint significantly reduces the resonant frequency of the cantilever structure, limiting its degrees of freedom in terms of force and vibration compared with the free structure.

### 4.3. Surface Stress Distribution and Magnetic Field Analysis

Since ME antennas realize electric–magnetic transformation through stress and strain mediation, mechanical and magnetic variables including deformation, stress and magnetic flux density are introduced to reveal the internal energy transfer law and interpret the mechanism accounting for performance differences among various antenna profiles. In the preceding analysis, we preliminarily explored the stress distribution and concentration regions. However, stress distribution analysis alone is insufficient to fully describe the operating characteristics of the antenna system. To gain a deeper understanding of the system’s dynamic behavior, particularly the impact of stress concentration effects on performance, further investigation into the displacement offset, stress, and magnetic flux density distribution under operating conditions is required.

**Figure 10:**
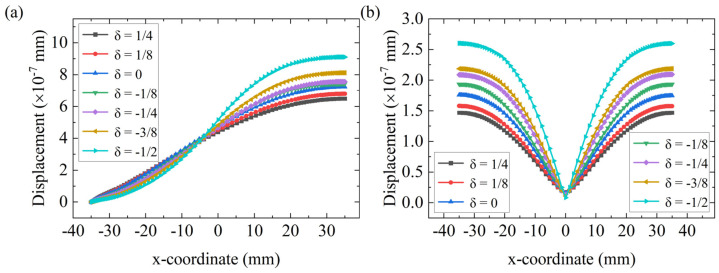
Displacement offset along the central axis: (**a**) Cantilever structure and (**b**) free structure.

Figure 10 shows the displacement offset distribution along the central axis of the upper surface of the BCB ME antennas under different control factors. As δ decreases, the displacement response of the antenna exhibits a significant change, reflecting changes in the vibration mode and structural stiffness. In the cantilever structure, the constraint at the fixed end induces significant nonlinear deformation, especially at smaller δ values, where the stress concentration effect leads to increased displacement. Specifically, a smaller δ reduces the equivalent stiffness of the structure, resulting in a sharp increase in displacement at the middle and the free end of the cantilever.

In contrast, the displacement distribution of the free structure is more symmetric, and its vibration mode is relatively uniform. Nevertheless, as δ decreases, the structural stiffness is reduced, leading to an overall increase in displacement. This indicates that the control factor significantly influences the mechanical response and resonant characteristics of the antenna by adjusting the geometric configuration and stress distribution. The relationship between stress distribution and displacement offset reveals the profound impact of structural constraints and material nonlinearity on antenna performance. Therefore, by tuning δ, the vibration characteristics of the antenna can be effectively optimized.

**Figure 11:**
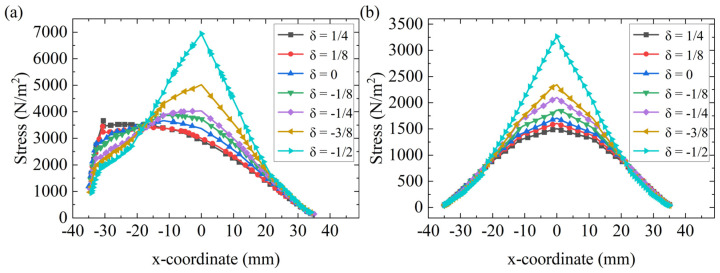
Stress distribution along the central axis: (**a**) Cantilever structure and (**b**) free structure.

Figure 11 shows the stress distribution along the central axis of the upper surface of the antennas under different control factors. As δ decreases, the stress distribution of the antenna exhibits significant changes. Particularly in the cantilever structure, stress concentration occurs at the free end, indicating that stress intensifies as the structural flexibility increases. Under different boundary constraints, the stress reaches its maximum value at the narrowest width location. As δ continuously decreases, the maximum stress gradually increases. It can be observed from the figure that as δ decreases, the peak stress of the cantilever structure shifts toward the free end, reflecting the significant influence of boundary effects on the antenna vibration. In contrast, the free structure, being unconstrained at both ends, exhibits a more uniform stress distribution. However, as δ decreases, the peak stress consistently remains at the central axis, revealing the interplay between reduced stiffness and nonlinear deformation.

**Figure 12:**
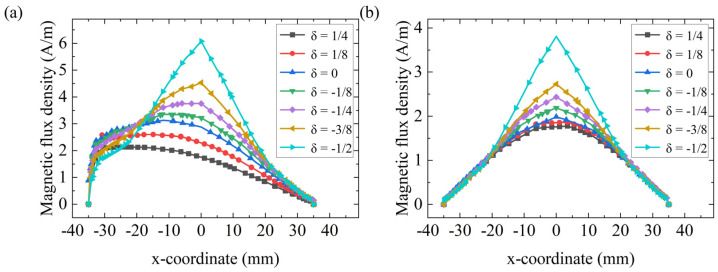
Magnetic flux density distribution along the central axis: (**a**) Cantilever structure and (**b**) free structure.

Figure 12 illustrates the magnetic flux density distribution along the central axis of the mid-surface of the magnetic layer of the BCB ME antenna under different control factors δ. Under different boundary constraints, the magnetic flux density along the central axis first increases and then decreases. Notably, the distribution of magnetic flux density along the central axis exhibits a trend consistent with the stress distribution, showing a concentration phenomenon. This indicates that in the radiation mechanism of the ME antenna, stress variations influence the magnetic flux density, thereby affecting the nonlinear response and ME coupling effect of the system. By adjusting δ, the stress distribution can be modified, enabling an enhancement of the ME coupling effect even when the overall volume is reduced. This provides a theoretical basis for subsequent antenna design and optimization.

### 4.4. Far-Field Radiation Characteristics Analysis

**Figure 13:**
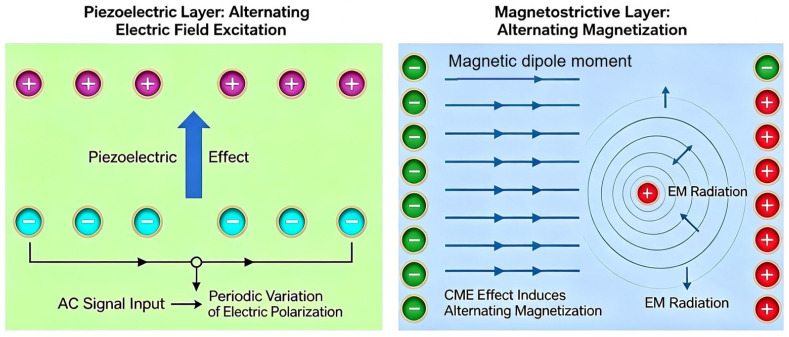
Far-field radiation mechanism of ME antennas.

Far-field radiation characteristics (radiation pattern) reflect the ability of the antenna to radiate EM waves into free space, which act as the ultimate evaluation indicators for practical wireless applications. For far-field radiation calculation, the surface current density and surface magnetic current density on the near-field spherical surface surrounding the antenna are adopted as radiation sources and imported into the RF Module built into COMSOL Multiphysics. This module integrates relevant mathematical models via its built-in compiler. Alternatively, the equivalent magnetic dipole radiation model can be utilized for analysis. These two approaches share essentially the same physical principle. For an ME antenna, radiation arises fundamentally from the periodic variation of magnetization intensity within the magnetostrictive layer induced by the CME effect. This alternating magnetization can be physically equivalent to a set of time-harmonic oscillating magnetic dipoles [17]. The specific radiation mechanism is shown in Figure 13. In the present work, this equivalence method is employed to analyze the far-field radiation of the BCB ME structure. The equivalent magnetic dipole moment is derived from the magnetization intensity extracted from finite element simulations. When the magnetic dipole moment oscillates resonantly, EM waves are radiated into free space. In the far zone where the observation distance greatly exceeds the antenna size and operating wavelength, the far-field electric and magnetic fields can be described by the classical magnetic dipole radiation formulation, namely, Equation (4), which incorporates variables associated with mechanical vibration. Superposition based on Equation (4) allows efficient prediction of antenna radiation performance and avoids computationally expensive full multi-physics coupled simulations. Detailed derivations of the far-field expressions and magnetic dipole moment are available in our previous work [33].

(4) Eϕm=ωβ2pm4πsinθ−1βr+j(βr)2e−jβrHθm=β2ωpm4πηsinθ1βr+j(βr)2+1(βr)3e−jβr

where *β* is the wavenumber of the EM wave in free space, pm is the magnetic dipole moment induced on the surfaces of the magnetostrictive layer, *r* is the radiation distance, and *η* is the wave impedance.

In the present study, magnetic flux is acquired from numerical simulations. Parameter *δ* controls the contour of the Bessel-curve boundary. Different values of *δ* correspond to ME antennas with varied cross-sectional profiles, as illustrated in Figure 2. Each contour determined by *δ* yields a distinct magnetic flux distribution, which further determines the magnitude and distribution of the equivalent magnetic dipole moment pm. In this way, the effect of parameter *δ* is introduced into Equation (4) through the magnetic dipole moment pm, and thus, this formula can characterize the far-field radiation performance of BCB antennas.

From the above expressions, it can be seen that the far-field radiation of a magnetic dipole exhibits pronounced directivity, with the radiation intensity proportional to sinθ. The radiation intensity reaches its maximum when the observation direction is perpendicular to the magnetic dipole moment (θ=90°) and drops to zero along the axial direction of the magnetic dipole moment (θ= 0° or 180°). Consequently, the radiation pattern of a magnetic dipole presents a typical “donut” shape.

**Figure 14:**
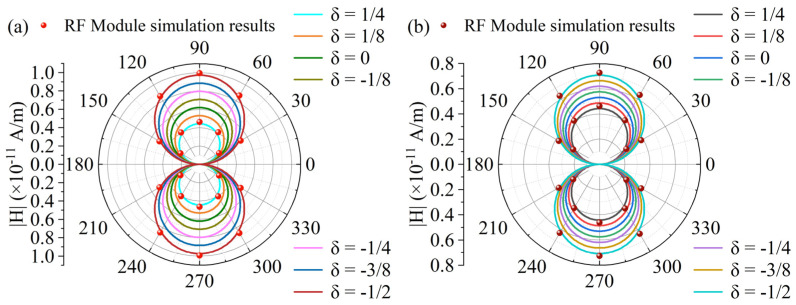
Far-field radiation patterns: (**a**) Cantilever structure and (**b**) free structure.

The calculated magnetic dipole moment is substituted into the radiation field model of the magnetic dipole and simulated using MATLAB 2021. Figure 14 shows the radiation field distributions of the BCB ME antenna under different control factors δ, corresponding to the far-field patterns of the cantilever structure and the free structure, respectively. Herein, the scattered dots denote results calculated by the RF Module, while the solid lines correspond to predictions from the equivalent magnetic dipole model. Excellent quantitative agreement can be observed between the two sets of data, which further verifies the reliability of the proposed model. The figure illustrates the influence of different δ values on the radiation field of the antenna, where each curve represents the distribution of magnetic field intensity under a specific control factor. It can be observed from the figure that as δ increases, the symmetry and directivity of the radiation field in the polar coordinate system exhibit significant changes. In the cantilever structure, smaller δ values (e.g., δ = −1/8 and δ = 0) lead to enhanced magnetic field intensity in specific directions, indicating a significant impact on the antenna’s radiation pattern. As δ continuously decreases, the magnetic field gradually increases, reaching a maximum value of 0.91 × 10^−11^ A/m at δ = −1/2. In contrast, for the free structure, the magnetic field distribution is more uniform. As δ varies, the radiation pattern exhibits certain directional adjustments. Notably, under larger δ values, the radiation pattern of the antenna changes significantly, further demonstrating the regulatory role of structural stiffness and stress distribution on antenna radiation performance.

## 5. Conclusions and Limitations

### 5.1. Conclusions

This paper proposes a BCB ME antenna structure to meet the demands of miniaturization and integration in wireless communication systems. A nonlinear multi-field coupled numerical simulation model is established to systematically analyze its resonant frequency, stress distribution, magnetic flux characteristics, and CME coupling performance. The main conclusions are as follows:

(1) The BCB structure significantly alters the internal physical field distribution. Compared with conventional UCS antennas, the BCB ME antenna exhibits pronounced stress concentration and magnetic flux convergence during operation, providing a physical basis for the spatial reconstruction of magnetization modulation in the magnetostrictive layer.
(2) Geometric tuning effectively reduces the system’s resonant frequency. As the shape tuning factor decreases, the antenna’s resonant frequency exhibits a monotonic downward trend. Under the clamped boundary condition, the resonant frequency of the 70 mm-scale antenna decreases from 9 kHz to 7.2 kHz; under the free boundary condition, it decreases from 18.1 kHz to 11.1 kHz. This demonstrates that system low-frequency operation can be achieved solely through geometric configuration optimization without increasing material volume.
(3) The CME coupling capability and radiation performance are simultaneously enhanced. Contrary to the trend of resonant frequency, as the shape tuning factor decreases, both the CME coupling coefficient and the far-field radiation characteristics improve. Under clamped and free boundary conditions, the CME coupling capability is enhanced by 124% and 140%, respectively.
(4) The BCB design outperforms conventional UCS structures. When the shape tuning factor equals 1/2, the proposed design degenerates into a conventional UCS antenna, verifying model consistency. Comparative results demonstrate that the BCB approach exhibits superior low-frequency and strong-coupling potential at small scales, confirming the effectiveness of geometric tuning in optimizing ME antenna performance.

In summary, the BCB structure proposed in this paper provides a new design approach for achieving low-frequency, high-efficiency, and miniaturized ME antennas. The established multi-field coupled analysis model lays a theoretical foundation for further structural optimization and application studies.

### 5.2. Limitations

The model developed in the present study suffers from certain limitations, and further improvements will be implemented in future work:

(1) The epoxy bonding layer can be explicitly constructed in the model to reconstruct practical operating conditions and fully characterize its mechanical effect, although this approach will increase the computational cost.
(2) Damping effect parameters, which should be calibrated by experimental measurements, can be introduced to reduce the discrepancy between the theoretical resonant characteristics and experimental results.
(3) Finite element models can be coupled with equivalent circuit models to comprehensively capture the internal impedance of each functional material and other influencing factors arising from the circuit. In addition, further experimental investigations on the BCB antenna should be conducted to further verify its advantageous performance.

## Data Availability

The original contributions presented in this study are included in the article/Supplementary Materials. Further inquiries can be directed to the corresponding author.

## Supplementary Materials

The following supporting information can be downloaded at https://www.mdpi.com/article/10.3390/ma19153335/s1.

## Nomenclature

All symbols used in this study are defined in nomenclature.

l	Length of the BCB ME antenna (mm)
w	Width of the BCB ME antenna (mm)
B(τ)	Point on the Bézier curve at parameter τ
$P_{0-n}$	Number of control points of the Bézier curve
n	Degree of the Bézier curve
$w_{c}$	Adjustable dimensional parameter (mm)
δ	Shape control factor of the Bézier curve
$f_{r}$	Resonance frequency (Hz)
$\alpha_{CME}$	CME coefficient (A/V)
δH	Average AC magnetic field induced in the radiating layer (A/m)
δE	Average AC electric field induced in the piezoelectric layer (V/m)
γ	Geometric shape parameter
β	Wavenumber of the EM wave in free space (rad/m)
r	Radiation distance (m)
η	Wave impedance (Ω)
