# Supplementary material for: Design and Analysis of a Bézier Curve-Based Variable Cross-Section Magnetoelectric Antenna
Source: Materials (Basel). 2026 Aug 5;19(15):3335. doi: 10.3390/ma19153335 (PMC13468551; doi:10.3390/ma19153335)
Supplement: Supplementary file 1 [file materials-19-03335-s001.zip › materials-4450844-supplementary.pdf]

S1. Main Parameters of Materials

Table S1. Main material parameters of Terfenol-D.

Materials	M_s (A/m)	σ_s (MPa)	E (GPa)	λ_s (ppm)	χ_m	μ_r	$\tan\delta$
Terfenol-D	7.2×10^5	150	110	1000	12	13	0.004

Table S2. Main material parameters of PZT-5H.

Materials	d_{33} (10^{-12} C/N)	d_{31} (10^{-12} C/N)	ε_r	$\tan\delta$	s_{11} (10^{-9} F/m)
PZT-5H	370	-18.21	3400	0.002	24.49

S2. Boundary Conditions

For the cantilever structure, the mechanical boundary condition of the ME composite is fixed at one end and free at the other. Accordingly, the corresponding electrical and mechanical boundary conditions can be expressed as follows:

$$\begin{aligned} &x=-\frac{l}{2}:\sigma=0,\quad z=-\frac{h_p}{2}:V=0 \\ &x=\frac{l}{2}\quad \text{and}\quad y=\pm\frac{w}{2}:\mathbf{u}=0 \end{aligned} \tag{S1}$$

where h_p is the thickness of the piezoelectric layer. For the free structure, the mechanical boundary condition is free at both ends, expressed as follows:

$$x=\pm\frac{l}{2}, y=\pm\frac{w}{2}, z=\pm\frac{h_p}{2}:\quad \sigma=0 \tag{S2}$$

S3. Lumped Mass Method

Finite element analysis can simulate the dynamic response of complex structures in detail, fully accounting for the nonlinear behavior of materials and structures as well as complex boundary conditions. However, it is computationally intensive and time-consuming, especially when the resonance frequency is uncertain and a wide frequency range must be scanned. In contrast, the lumped mass method simplifies the mass distribution of a continuous medium into a set of lumped mass elements, providing the basic dynamic characteristics of the system without complex computations. Particularly for frequency response analysis, the lumped mass method can quickly estimate the system's resonance frequency. Therefore, in this subsection, the lumped mass method is adopted to preliminarily estimate the resonance frequency of the BCB ME antenna, thereby providing a reliable and efficient frequency sweep range for subsequent finite element simulations and improving computational

efficiency.

When the length of the ME antenna is much larger than its width and thickness, the internal displacement field $u(x,t)$ can be considered one-dimensional, expressed as $u(x,t)=\phi(x)q(t)$, where $\phi(x)$ is the mode shape function and $q(t)$ is the generalized coordinate. Under the combined action of an alternating electric field and a bias magnetic field, periodic coupled vibrations are generated inside the ME antenna, which can generally be approximated as simple harmonic vibrations. Taking the free-free boundary condition as an example, the mode shape function and generalized coordinate can then be written as follows:

$$\begin{aligned}\phi(x) &= \cos\left(\frac{2\pi x}{l}\right)\\ q(t) &= \Delta r\cdot\sin(\omega t+\theta)\end{aligned} \tag{S3}$$

where Δr is the displacement amplitude, ω is the angular frequency of the system, and θ is the phase angle. According to the relationship between displacement and velocity, the velocity distribution $v(x,t)$ of the system is:

$$\begin{aligned}v(x,t) &= \phi(x)\cdot\dot{q}(t)\\ &= \cos\left(\frac{2\pi x}{l}\right)\cdot\Delta r\cdot\omega\cos(\omega t+\theta)\end{aligned} \tag{S4}$$

As a continuous system, the total kinetic energy of the BCB antenna is obtained by integrating the kinetic energy of all mass infinitesimals. This integral is determined by the velocity distribution function over the entire domain of the system:

$$\begin{aligned}\Gamma &= \frac{1}{2}\int_{-l/2}^{l/2}\rho A(x)v^2(x,t)dx\\ &= \frac{1}{2}\dot{q}^2(t)\int_{-l/2}^{l/2}\rho A(x)\cos^2\left(\frac{2\pi x}{l}\right)dx\end{aligned} \tag{S5}$$

where ρ is the equivalent density of the ME antenna and $A(x)$ is the cross-sectional area. In simple harmonic vibration, kinetic energy and potential energy convert periodically into each other. Therefore, the maximum kinetic energy of the continuous system can be equated to the maximum kinetic energy of an equivalent single-degree-of-freedom (SDOF) spring-mass system:

$$\Gamma_{\max} = \frac{1}{2}M_{eq}(\Delta r\omega)^2 \tag{S6}$$

where M_{eq} is the equivalent mass of the system. Combining Eq. (S5) and Eq. (S6), the equivalent mass is obtained as follows:

$$M_{eq}=\int_{-l/2}^{l/2}\rho A(x)\cos^2\left(\frac{2\pi x}{l}\right)dx \tag{S7}$$

The potential energy of the continuous system can be expressed as follows:

$$U=\frac{1}{2}\int_{-l/2}^{l/2}EA(x)\left(\frac{\partial u}{\partial x}\right)^2 dx \tag{S8}$$

where E is the equivalent elastic modulus of the system. For a single-degree-of-freedom system, the maximum potential energy is:

$$U_{\max}=\frac{1}{2}K_{eq}\Delta r^2 \tag{S9}$$

where K_{eq} is the equivalent stiffness of the system. Using the condition that the maximum potential energies of the continuous system and the equivalent SDOF system are equal, the equivalent stiffness is derived as follows:

$$K_{eq}=\frac{\pi^2}{l^2}\int_{-l/2}^{l/2}EA(x)\sin^2\left(\frac{\pi x}{l}\right)dx \tag{S10}$$

Combining Eq. (S7) and Eq. (S10), the resonance frequency f_r of the system can be expressed as follows:

$$f_r=\frac{1}{2l}\sqrt{\frac{E}{\rho}\cdot\frac{\int_{-l/2}^{l/2}A(x)\sin^2\left(\frac{\pi x}{l}\right)dx}{\int_{-l/2}^{l/2}A(x)\cos^2\left(\frac{\pi x}{l}\right)dx}} \tag{S11}$$

From the Bézier curve equation in Eq. (3), the cross-sectional area $A(x)$ of the antenna can be obtained as follows:

$$\begin{aligned} A(x)&=2y(x)\cdot h \\ &=wh\left\{1+\left(\delta-\frac{1}{2}\right)\left[1-\left(\frac{2x}{l}\right)^2\right]\right\} \quad -\frac{l}{2}\le x\le\frac{l}{2} \end{aligned} \tag{S12}$$

Finally, substituting Eq. (S12) into Eq. (S11), the resonance frequency of the Bézier curve-based antenna is given by:

$$f_r(\delta)=\frac{l}{2l}\sqrt{\frac{E}{\rho}\cdot\frac{3\pi^2+(2\delta-1)(\pi^2+3)}{3\pi^2+(2\delta-1)(\pi^2-3)}} \tag{S13}$$

Eq. (S13) shows that, for a given material and fixed length, the resonance frequency of the BCB ME antenna is determined solely by the shape control factor δ , which preliminarily verifies the rationality of the design proposed in this paper. The foregoing derivation assumes free-free boundary conditions. For other boundary

configurations, the derivation process of the resonant frequency is analogous, while the adopted mode shape functions must satisfy the respective boundary restraints.
